# The Convergence of High-Consequence Livestock and Human Pathogen Research and Development: A Paradox of Zoonotic Disease

**DOI:** 10.3390/tropicalmed3020055

**Published:** 2018-05-30

**Authors:** Julia M. Michelotti, Kenneth B. Yeh, Tammy R. Beckham, Michelle M. Colby, Debanjana Dasgupta, Kurt A. Zuelke, Gene G. Olinger

**Affiliations:** 1 MRI Global, Gaithersburg, MD 20878, USA; kyeh@mriglobal.org (K.B.Y.); ddasgupta@mriglobl.org (D.D.); golinger@mriglobal.org (G.G.O.); 2 College of Veterinary Medicine, Kansas State University, Manhattan, KS 66503, USA; trbeckham@vet.k-state.edu; 3 Institute of Food Production and Sustainability, National Institute of Food and Agriculture, United States Department of Agriculture, Washington, DC 20250, USA; Michelle.Colby@nifa.usda.gov; 4 Strategic Biosecurity and Biocontainment Facility Management Consultant, Kurt Zuelke Consulting, Lenexa, KS 66220, USA; kurtzuelke@gmail.com

**Keywords:** biosafety, biosecurity, BSL-3-Ag, high-consequence pathogen research, zoonotic disease

## Abstract

The World Health Organization (WHO) estimates that zoonotic diseases transmitted from animals to humans account for 75 percent of new and emerging infectious diseases. Globally, high-consequence pathogens that impact livestock and have the potential for human transmission create research paradoxes and operational challenges for the high-containment laboratories that conduct work with them. These specialized facilities are required for conducting all phases of research on high-consequence pathogens (basic, applied, and translational) with an emphasis on both the generation of fundamental knowledge and product development. To achieve this research mission, a highly-trained workforce is required and flexible operational methods are needed. In addition, working with certain pathogens requires compliance with regulations such as the Centers for Disease Control (CDC) and the U.S. Department of Agriculture (USDA) Select Agent regulations, which adds to the operational burden. The vast experience from the existing studies at Plum Island Animal Disease Center, other U.S. laboratories, and those in Europe and Australia with biosafety level 4 (BSL-4) facilities designed for large animals, clearly demonstrates the valuable contribution this capability brings to the efforts to detect, prepare, prevent and respond to livestock and potential zoonotic threats. To raise awareness of these challenges, which include biosafety and biosecurity issues, we held a workshop at the 2018 American Society for Microbiology (ASM) Biothreats conference to further discuss the topic with invited experts and audience participants. The workshop covered the subjects of research funding and metrics, economic sustainment of drug and vaccine development pipelines, workforce turnover, and the challenges of maintaining operational readiness of high containment laboratories.

## 1. Introduction

**Figure 1:**
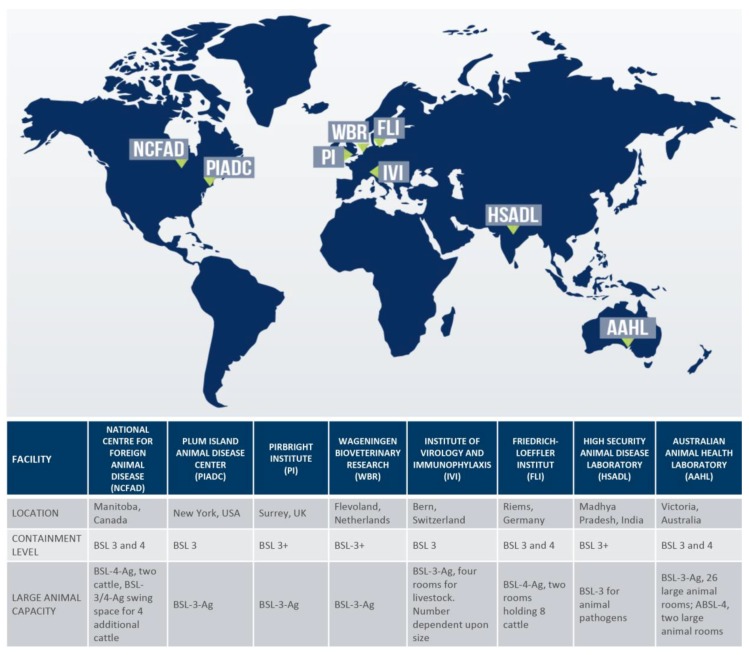
Worldwide map illustrating the locations of high-containment laboratories that can accommodate agricultural livestock. There are currently four BSL-3-Ag facilities, one BSL-3 for agricultural studies (HSADL), and three BSL-4 facilities [1]. The number of research animals that can be accommodated at one time is noted in the table when available.

High-consequence pathogens are viruses and bacteria that have epidemic or biothreat potential where an effective therapeutic or vaccine does not exist. Animal studies on high-consequence pathogens affecting the agricultural livestock industry must be performed in biosafety level 3 Agriculture (BSL-3-Ag) or BSL-4 facilities that can accommodate large animal research (Figure 1) [1]. BSL-3-Ag facilities are unique to agriculture because of the necessity to protect the environment from the biosecurity and economic risks that could occur from an accidental release of a high-consequence pathogen. In these facilities, the barriers for each room serve as the primary biocontainment, so they require almost all the features used for BSL-4 facilities in addition to the standard BSL-3 facility requirements [2]. Facilities that are configured to carry out research studies on large livestock (e.g., cows, horses, and sheep) are equipped with oversized equipment, such as downdraft procedure tables and autoclaves. In addition, these facilities have specialized caging areas and utilize advanced handling techniques for animals, such as horses, that may be stressed in the high-containment laboratory environment. The challenges are that BSL-3-Ag and BSL-4 facilities are expensive to build, operate and maintain and require a highly trained and consistent workforce. Facility operations also require robust biosafety and biosecurity programs and personnel reliability programs that assure safety and security in the workplace and surrounding areas. For example, high-consequence pathogen research on the *Aphthae epizooticae* virus, which causes foot-and-mouth disease (FMD), is currently geographically isolated in the U.S. at the Plum Island Animal Disease Center facility in New York [3]. To date, there are a limited number of high-containment facilities in countries such as Brazil, India, Guinea, Sierra Leone, and Liberia that have recently experienced high-consequence pathogen outbreaks. The lack of laboratory support in these settings means that international collaborations are critical for successfully responding to the next emerging or re-emerging disease outbreaks that may affect their populations and economies.

**Figure 2:**
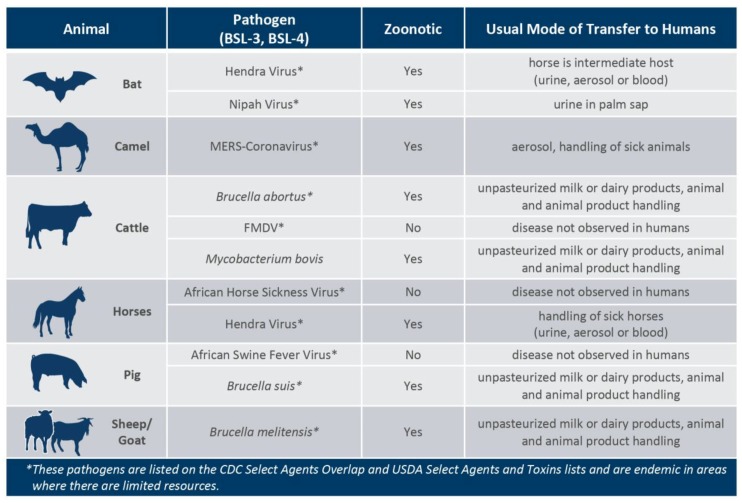
Both livestock and wild animals are impacted by zoonotic pathogens that require specialized BSL-3 or BSL-4 facilities. Several of the agricultural high-consequence pathogens discussed during this workshop are on the Select Agent list and many are also are infectious to humans (zoonotic).

It has been shown that many emerging high-consequence pathogens for agriculture and public health originate in wildlife, including wild pigs, birds, and bats. Additionally, some pathogens can be carried asymptomatically by livestock and may or may not be infectious to humans (Figure 2) [4,5,6,7,8]. As people continuously migrate to new areas, particularly near recently de-forested areas, closer proximity to insect or animal populations carrying pathogenic viruses and bacteria increases the risk of disease to those not previously exposed. In response to these challenges many international health organizations are promoting the adoption of the One Health concept. The U. S. Centers for Disease Control and Prevention defines One Health as a ‘collaborative, multi-sectoral, and trans-disciplinary approach (working at the local, regional, national, and global levels) with the goal of achieving optimal health outcomes recognizing the interconnection between people, animals, plants, and their shared environment’ [9]. The One Health concept takes this interface between human, animal, and ecological factors into account and aims to formulate more efficient and fruitful interdisciplinary research and development studies. However, divergent stakeholder interests and regulatory environments associated with agricultural, environmental, and public health disciplines present a potential obstacle for funding comprehensive One Health research programs. For example, while veterinary animal health research can address the potential public health implications of zoonotic animal pathogens, researchers must often strive to minimize potential adverse effects on livestock production or market access restrictions on livestock and animal products that impact traditional agricultural stakeholders.

In the One Health model, human, animal, and environmental health research funding agencies need to communicate and collaborate to leverage resources that may be required by each discipline [10]. For example, the human and agriculture drug and vaccine development pipelines have similar lengthy processes to develop and validate products. However, there are unique regulatory requirements for drug product production and testing by each country in addition to international rules and guidelines. Additionally, for the food industry there are extensive regulations on if, how, and when pharmaceuticals or biologics can be used on animals destined for human consumption. It is especially difficult to get drug development projects funded that are targeted to medical counter-measures for those biothreat agents that do not have a high enough return on investment (ROI) to justify commercial and private investment. In this case, national government funding is required to provide grants and other financial support for both research and development [11]. There are also infrastructure limitations, as for Select Agent pathogens that may also be zoonotic, where either BSL-3-Ag or BSL-4 facilities are required for the critical efficacy testing phases of translational research and clinical trials for both vaccines and drug products. As mentioned earlier, lack of high containment laboratory capacity, in addition to economic conditions in low income and low-middle income countries, typically means there is a dependence on international aid with large efforts to build sustainable in-country capacity for research and development. To take advantage of a scientific discussion forum at a professional society conference attended by experts, we designed a workshop to cultivate awareness and discussion on these current topics.

## 2. Workshop Design, Structure, and Topics

Scientific conferences, especially those presented through professional organizations such as the American Society for Microbiology, are important forums to present new findings to peers and the professional community.Besides the plenary talks that highlight a conference, additional information is exchanged during oral and poster presentations, and interactive discussions can take place in roundtables and workshops. The variety of discussions offer opportunities for face to face interaction, which is often a catalyst for new collaborations, especially among people from varying disciplines. The in-person meetings, whether one-on-one or in groups, reinforce professional relationships, friendships, and build comradery. These interactions are essential in building a foundation of mutual respect and trust—and are vital in advancing complex dialogs in areas such as research, biosafety and biosecurity [12]. Our approach for designing this workshop was to craft a relevant, moderated discussion that would include expert panelists. After defining our topics and objectives, our team developed questions for discussion with the objective of collaborating to publish the findings. Before our workshop, we invited attendees and participants to join an online forum where they could learn about our work, see the background of our experts, and otherwise engage by posting questions. A Workplace by Facebook was set up as a closed, private group where participants were invited and given the option to join. A small group including the moderator and expert panelists convened via email and short conference calls to develop the specific discussion topics for the two-hour workshop that included questions from the audience. Overall, this approach is defined, repeatable, and contributes to scientific knowledge in a rapid and timely manner. With the advent of social media tools and apps, such activities and discussions that continue to take place at conferences increase the awareness of the event and catalyze innovative discussions.

**Figure 3:**
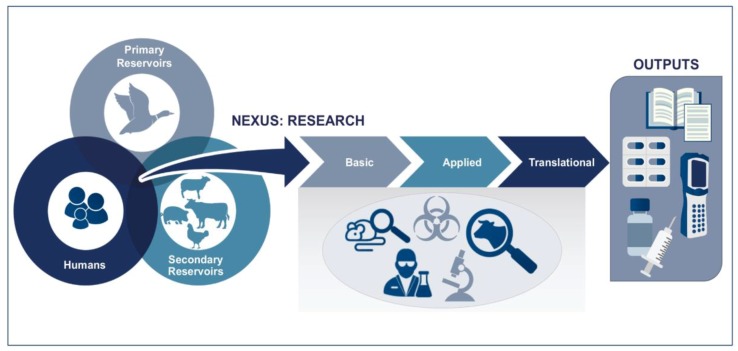
Diagram showing the intersection between animal health, human health and the ecological factors that must be considered when prioritizing research topics. The basic, applied, and translational scientific areas involve concerns of biosafety and biosecurity along with the operational considerations when choosing specific small or large animal models.

The workshop questions were designed to highlight areas where the animal and human product development streams might converge so that the length of time for each stream could be shortened, leading to life-saving vaccines being available sooner. For example, when studying high-consequence pathogens, much of the early research work is done in traditional BSL-3 or BSL-4 facilities. It is during the translational and clinical trial stages that the BSL-3-Ag or BSL-4 agricultural facilities are needed to carry out relevant animal model studies. The workshop questions also addressed ongoing efforts to improve the process of bringing a vaccine for a newly-emerging pathogen from virus/bacteria discovery to commercialization in the agriculture sector. Figure 3 illustrates the typical One Health concept, where research on primary reservoirs (ecological and epizootiological study), secondary reservoirs (wild or domesticated animals), and human health is overlapping and the basic and applied areas of development overlap. It is at the early stages when the disease is first diagnosed (collecting samples for pathogen isolation/identification) and later stages where treatments are tested in animals, that the activities diverge.

Our expert panelists (Tammy Beckham, D.V.M, Ph.D., Michelle Colby, D.V.M, M.S., and Kurt Zuelke, D.V.M, Ph.D.) are distinguished veterinarians with experience in administration, consulting, management, research, and policy from academia, government, and industry. Each panelist has extensive expertise in researching and managing complex agricultural projects involving high-consequence pathogens.

The topics that were provided ahead of time to the panelists were as follows:

- Priority zoonotic research gaps, especially for projects requiring BSL-4 large animal (BSL-3-Ag or BSL-4); potential Select Agent challenges.
- The operational challenges of maintaining BSL-3-Ag and BSL-4 laboratories for livestock research.
- Workforce development challenges for sustainment of safe and secure research capabilities.
- Novel business and financial approaches for long-term program sustainability.

## 3. Discussion—Moderator and Panelists

Our moderator opened the discussion with introductions and remarks. The workshop was transcribed with the ten questions followed by a synopsis of the answers and discussion. The panelists were given time to comment, discuss the topic with each other, and to answer questions from the participants at the end of the formal questioning. The order of the questions and synopsis was edited for presentation in this article.

### 3.1. In the Area of Detection and Response to Emerging Pathogens, What Problems Keep You up at Night?

The interface between human health and animal health is complex and work on one without considering the other can have unintended consequences. For example, a recent outbreak of avian influenza in Iran lead to culling of chickens using standard public health practices. This led to an egg shortage that caused a significant increase in the price of eggs, which helped contribute to political unrest in the fall of 2017 [13]. Also, regional experience working in Southeast Asia on projects involving FMD biosurveillance revealed that it is important to follow the regional livestock production, supply chain, and animal markets to inform and understand the epidemiology of FMD and other disease outbreaks. An outbreak of disease that is blamed on a widely-used food source will be problematic and there is a need to respond rapidly. Reporting the presence of FMD in cattle on one commercial farm has the result of halting exports of milk and meat for the entire country [7]. The nexus between social structure, economy, safe food, and human health underscores the need for scientists to come together in the event of an outbreak and be prepared to solve the problem, while avoiding scare tactics. When these types of outbreaks occur in areas of unrest, or with high mobility of people and animals, there is a possibility of a zoonotic event that we cannot respond to with a vaccine or diagnostic test in an effective manner. In order to quickly identify new emerging pathogens during outbreaks we need rapid, accurate, and validated point-of-care diagnostics. If we can effectively predict disease emergence based on ecological and sociological factors we can be prepared with stockpiled vaccines or approved methods and management strategies to rapidly generate and disseminate diagnostic tests and countermeasures that minimize potential impacts of these diseases. There would also be value in using advanced epidemiological models and machine learning if we can incorporate biosurveillance data from decentralized sites to a central data management source.

### 3.2. What Are the Existing Research Gaps that Impact Both Human and Animal Health? Is There Any Low-Hanging Fruit?

In the field of veterinary comparative medicine deep sequencing is a valuable tool that is becoming more accessible. Studying the host interactions during virus infection is also critical, especially in understanding how a host can maintain infection asymptomatically or become a carrier. As an example, Hendra virus infection in bats has a different clinical course than in the horse and sequencing studies on isolates from both species would help determine the host differences. Various funding institutions would need to work together to initiate this type of study and it would benefit from using an internet-based ‘community of practice’. Current examples of this type of collaboration are the Global African Swine Fever Research Alliance and the Global Foot-and-Mouth Disease Research Alliance. Improved collaboration and sharing of reagents/standards for diagnostics and biosurveillance would be an economical method for improving efficiency. Also, sharing of data and data analysis tools is important for the ability to perform risk assessments during an outbreak.

### 3.3. How Are the Human and Agriculture Development Pipelines Similar or Different? What Are the Drivers?

**Figure 4:**
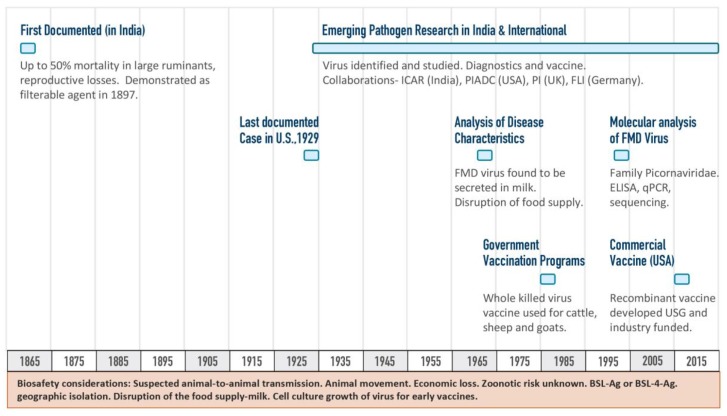
Timeline of the research and development of the foot-and-mouth disease (FMD) vaccine. This timeline shows highlights from early FMD discovery (focusing on India) to the licensure of a livestock vaccine [7,14,16]. The Friedrich-Loeffler Institut in Riems Germany (Figure 1) is the location of the first research work done on foot-and-mouth disease.

The drug development pipelines are similar in that they are highly regulated with the required basic steps of research, development, toxicology, clinical trial, and post-approval testing. There are not many products that make it through the medical countermeasures (MCM) pipeline especially for diseases that affect agriculture only. The current FMD vaccine licensed in 2012 was successfully developed without an outbreak as a primary driver (Figure 4). The Departments of Homeland Security and Agriculture funded the agriculture vaccine research, while the Department of Health and Human Services funded human countermeasure research, production, and procurement (including the strategic national stockpile), in part through Project BioShield [14].

**Figure 5:**
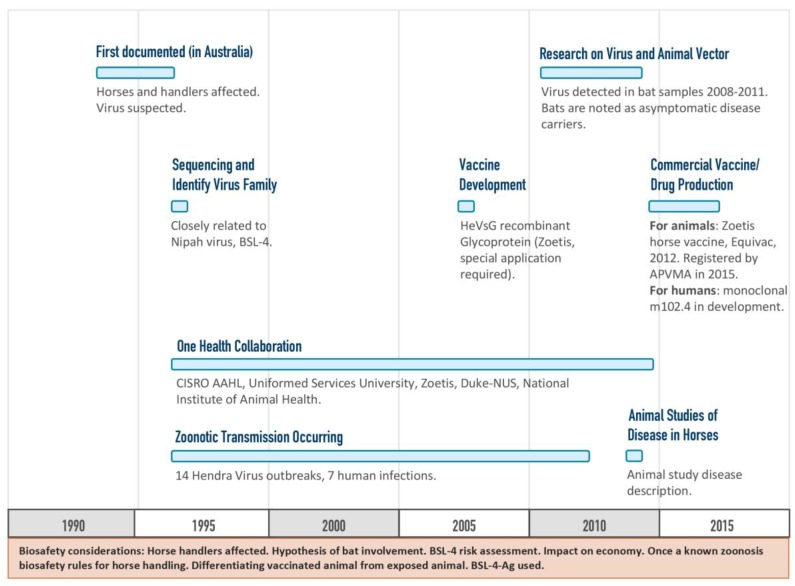
Timeline of the research and development of the Hendra virus vaccine. This timeline shows highlights from the initial Hendra virus disease discovery to the licensure of a vaccine for livestock [4,15,17].

The Hendra vaccine was developed among partners with a mutual aim to prevent the spread of disease to people by vaccinating horses and thereby eliminating the horse as an intermediate host for the Hendra virus (Figure 5) [15]. Development of the Hendra vaccine provides a case study whereby a vaccine against a BSL-4 agent was approved and is in commercial use. The type of funding model used for the Hendra vaccine development was non-linear but still forward-progressing, and incorporated a One Health approach that utilized information from multiple tracks of ongoing research.

### 3.4. In Order to Diagnose an Outbreak Pathogen Early in the Process, Pen-Side Diagnostics Are Believed to Be Helpful. Has There Been Good Progress with Pen-Side Diagnostics?

There are some pen-side diagnostic tools available for agriculture but none are approved for use during an outbreak. Often we know that science outpaces policy especially in areas where there is impact to trade and economy. For example, it must be determined who has the authority to release test results and what the implications are of releasing an assay result, especially if the false positive or negative rate of the assay is high. Human point-of-care is more advanced than pen-side. This also raises the question of the absence of a centralized system for real-time data reporting; currently there is no capability to bridge this gap especially in the livestock sector. If we are going to move forward with pen-side diagnostics, then the policies that support their use must be prioritized along with incentives for data sharing and reporting.

### 3.5. What Would a Dream Biosurveillance System Look Like? What New Investments in Data Management Tools Should We Make Now to Get a Large Return?

The ideal biosurveillance system should have human and animal data together, commercial industry participation, be cloud-based, secure, and offer real-time access. In this case it would be necessary to have a governance board to make sure that data is shared appropriately and protected. It should have an easy-to-use dashboard with the option for hospital and diagnostic labs to access reports based on various key attributes (animal ID, patient ID, location, and disease). It should also provide incentives for all users that participate and provide data in the system by offering real-time data that provide situational awareness and the ability to make decisions quickly. The National Antimicrobial Resistance Monitoring System (NARMS) and AgConnect are good examples of where data sharing is currently being implemented [18].

### 3.6. What Do You Think Is the Role of Different Funding Sources? Public-Private Partnerships Are an Option but It Is Hard to See How These Will Evolve Moving Forward. Will the Funding Agencies Ever Have Development Groups?

A better understanding of the epidemiology, pathogenesis, and ecology of these diseases is needed and the animal-vector-human interface needs to be studied and understood in order to break the transmission cycle. Multi-disciplinary collaboration to address key challenges in this area were supported by the joint Department of Homeland Security/NIH Fogarty International Center ‘Research and Policy for Infection Disease Dynamics’ but the program is no longer active. In the future, MDM development efforts will need to be looked at for return on investment (ROI) metrics. Research in the agriculture sector has historically been government-funded, so the systems are not well set up for business-level analysis of their programs. However, public and private partnerships have always been important to the Department of Homeland Security Science & Technology Directorate (DHS S&T) and the USDA, Agricultural Research Service at Plum Island (USDA-ARS). The licensing of a new FMD vaccine in 2012 resulted from a successful public-private partnership including DHS S&T, USDA-ARS, and multiple industry partners. Other products are also in development via Cooperative Research and Development Agreements. Since we cannot vaccinate for something that we do not know is coming there is a need to develop therapies quickly (e.g., immunotherapeutic) to respond to disease ‘X’, as listed on the 2018 WHO Blueprint of Priority Diseases list [19]. If it is necessary to bring in private investors, then they will demand metrics so that ROI analysis is possible. Rabies is an example of a success story where the ROI was high due to the commercial demand for rabies treatments and both animal and human vaccines. The involvement of public or private investment for philanthropic reasons is one area where funding is currently increasing for diseases where the ROI may be sub-optimal. For example, the Pirbright Institute, the Bill and Melinda Gates Foundation and the Wellcome Trust all fund research initiatives in this area. Although philanthropic organizations are donating money they still require data that enable measurement of the success of specific initiatives (e.g., disease reduction, child health improvement). It is also important to keep the laboratory facilities side in mind. There is currently a 2-year wait for an Ebola vaccine efficacy study in the non-human primate (NHP) model. Large livestock animal (BSL-4-Ag) facilities may also have the capability to run NHP studies and could perhaps be used for this purpose. As illustrated with the development of the Hendra vaccine, the existing large animal BSL-4 facilities provide infrastructure and specialized comparative medicine and animal model expertise that could be effectively leveraged to develop and validate alternative animal models (e.g., ferret or swine) that could hasten development and regulatory approval for medical countermeasures against high-consequence pathogens. Operating and maintaining a BSL-4 agricultural facility with 24-7 capability to perform comparative animal and vaccine efficacy studies is expensive and requires a consistent and highly-trained workforce. Increasing the opportunities to make these facilities available to additional One Health partners could be an option to increase cost-effectiveness in operations and global utilization of this limited and highly specialized resource. A good example of this is the BSL4ZNet initiative that is designed to increase collaboration between high containment laboratory experts [20]. BSL4ZNet is funded by the Canadian Safety and Security Program and members include the international BSL-3-Ag and BSL-4 facilities listed in Figure 1 (NCFAD, AAHL, FLI, PI and PIADC). One major aim of this initiative is to enable knowledge sharing by producing a catalog of BSL-4 training opportunities, organizing a laboratory exchange program, and hosting presentations by subject matter experts.

### 3.7. ROI Is Not Always Reported for Basic Academic or Government-Funded Research but Commercial Companies Require It. Are You Seeing This as a Request in the Area of Veterinary Vaccine/Therapeutic Pipeline Development?

There is no inherent requirement for ROI reporting by private institutes and non-governmental organizations but, as an example, the Bill and Melinda Gates Foundation does look at the outcome and measures of success. A paradigm shift may be needed for this type of reporting by academically-trained researchers. It will also be useful to put quality assurance processes in place early, so that if the research progresses to product development, it will be useful as part of any subsequent regulatory data portfolio. There is now a marketing and business lens across many early research efforts and the quality system must be pre-positioned for the target selection and animal study approach. The Australian Animal Health Laboratory (AAHL) in Geelong recently introduced additional quality assurance procedures along with flexible overnight work shifts, when required for particular disease models, to enable the comparative medicine staff running animal studies additional time to meet the demands of using good laboratory practice (GLP). Partnering is key and establishing a ‘community of practice’ is necessary to effectively advance research on high-consequence pathogens from discovery to product development and clinical testing. It is important for researchers to lead their science and own their part, but also be willing to share. This concept of a scientist (and their lab team) working as an integrated member of a scientific ‘community of practice’ is perhaps a paradigm shift away from traditional academic models where individual principal investigators work exclusively on their respective academic or government research programs. With government-funded research there is a transition occurring where stakeholders want demonstrable commitment towards achieving their strategic objectives and value from their investment. There is a need to report quantifiable results of research outcomes and measure the impact and ‘value’ of research beyond publications. For example, it is easy to quantify the impact of a specific medical countermeasure but it is difficult to assess the value of the incremental, iterative research that is required to develop vaccine technology.

### 3.8. For Human MCMs, DOD and HHS Have Provided Funding to Get Projects through the ‘Valley of Death’ and Supported Manufacturing Capabilities. However, Development of MCM Presents Physical and Regulatory Risk to Manufacturing of Other Products. How Would You Recommend Bridging This Gap for Agriculture Research? 

For MCM the ‘valley of death’, a gap between bench research and clinical application, is often subsidized by national government efforts [14]. HHS’s Biomedical Advanced Research and Development Authority (BARDA) administers funding for Project BioShield, which supports human drug/vaccine/diagnostics efforts by bridging the ‘valley of death’ for products deemed critical for protecting public health from a biological attack. However, no similar line of funding exists on the animal health side. Many of the diseases of concern to U.S. livestock are endemic in other parts of the world and target product profiles are similar despite differences in industry and animal husbandry. The international demand for vaccines, drugs and biologics that would also benefit the U.S. increases the ROI for these products, incentivizing earlier investment from industry partners. While this does not completely eliminate the ‘valley of death’ for animal health countermeasures, it creates additional opportunities for public-private partnerships. In addition, developing and manufacturing countermeasures for high-consequence pathogens may require specialized facilities, permits and licenses—all of which add additional financial burden to private industry. This increases the importance of ROI. The research and development history involving Hendra and Nipah viruses serves as an example of where ROI is critical. The Australian government’s willingness to require equine vaccination to protect the human population created a significant enough market potential to stimulate the pursuit of a Hendra vaccine, while a similar vaccine for Nipah has never been realized, likely due to economic factors rather than scientific feasibility [21].

### 3.9. Do We Have the Technology and Workforce to Address the Challenges that Have Been Discussed?

There is a global shortage of highly trained and specialized veterinary and technical support staff capable of leading and performing BSL-3-Ag and BSL-4 large animal studies involving high-consequence pathogens. This shortage is compounded by high turnover and low retention of these specialized professionals due to the inconsistent on-again off-again nature of project funding combined with the high workloads and safety demands inherent with these types of projects. One potential solution to this capability shortage could be to share animal study workload across multiple BSL-3-Ag/BSL-4 labs that partner as a network and then collectively maintain a critical mass of staff that could move around on projects across this network.The students coming out of veterinary school may need additional training and potentially another advanced degree (e.g., Ph.D.) in order to fully advance in One Health research-based careers. For this to be possible, there is a need to lower veterinary student debt and also introduce students to the variety of rewarding research careers that are available in the government sector. At the same time we need to leverage the availability of qualified students that can further train to work in BSL-3-Ag and BSL-4 facilities. For example, there are DHS-funded fellowships that will be used to target training for the upcoming National Bio- and Agro-Defense Facility (NBAF) workforce. In addition, the USDA supports high containment training through fellowships and workforce development initiatives.

### 3.10. How Can We Better Position Ourselves for the Next Emerging or Re-Emerging Viral or Bacterial Pathogen Outbreak?

The ecology of disease emergence refers to the study of where in the environment insects, animals and humans interact and zoonotic transmission occurs. In looking at this field, there is an opportunity for epidemiologists and ecologists to work together on projects studying urbanization and social sciences. When people move to new, previously-uninhabited areas, significantly more vector and reservoir exposure occurs—especially in tropical areas. There is an entire new range of science for the ecology of disease evolution that should be explored. For example, NBAF, the new BSL-4 agricultural facility for large animal research and vaccine/therapeutic development that is currently under construction in the U.S., will bring opportunities for new R&D strategies. There is also a challenge in that U.S. government federal funding needs to be incentivized and that new resources will need to be leveraged along with existing sources [22]. The One Health concept brings together human and animal funding sources and it should be robustly promoted. On the applied side and for diagnostics the alignment of federal government funding is important. Government agencies, such as the Office of Science and Technology Policy, DHS, USDA, HHS, and the Department of Defense, that provide joint funding are good sources but they must be aligned for One Health type projects. Joint requirements of funding would help to establish communications and allow funding sources to include both basic and applied research. The National Institutes of Health ‘Biodefense and Emerging Infectious Diseases’ program is a good example of how we can prioritize One Health issues.

## 4. Workshop Findings

The overall concept for this workshop was to begin to understand and address the paradox that viewing animal, human, and arthropod disease research as separate, discrete activities, is confounded by the fact that the evolution and transmission of zoonotic diseases often occurs simultaneously and concurrently between multiple hosts. The discussion highlighted the One Health concept, biosafety and biosecurity readiness, and how biosurveillance can help prevent the spread of an emerging or re-emerging pathogen to densely populated areas, especially in low income countries.

The following themes emerged during the course of this workshop.

On prioritizing animal and zoonotic disease research and related drug pipeline projects:

➢ Collaboration between government funding agencies is needed to facilitate the study of both agriculture and public health issues in one project because it will mitigate funding disparities between agencies and ensure that there are no air gaps between animal health and public health research.
➢ Commercial and government groups should work together to fund vaccine development for diseases that can impact both animal and human health. A successful example is the Hendra virus vaccine development.
➢ Ideally, biosurveillance for emerging and re-emerging pathogens and the ability to share the data should be real-time and cloud-based.
➢ It is important to find a globally-accepted mechanism to promote data sharing for biosurveillance efficiency and to enable rapid notification of stakeholders in the event of a threat to public health.
➢ A challenge to real-time data sharing is that agriculture issues resulting in restrictions on live animal or animal product exports can affect the economics of countries and data in these situations must be well vetted prior to public release.
➢ We need to be able to respond quickly to disease ‘X’, which may be caused by a previously-unknown pathogen. In some disease outbreak scenarios social unrest is possible if vaccines, diagnostics, and therapeutics are not available.

On the regulatory aspects of drug and vaccine development for animals:

➢ Private and non-traditional research funding sources are becoming increasingly relevant. All funding sources are looking for scientific data that includes metrics that enable ROI measurement.
➢ Animal versus human vaccine and drug pipelines have very different regulatory requirements and it would be useful to consider these together as in the One Health model.

On biocontainment workforce development and consistency:

➢ Veterinarians and medical doctors should be extensively trained in One Health topics and take comprehensive coursework in zoonotic disease biology.
➢ Debt incurred due to high tuition costs is a significant consideration for graduating veterinarians. Tuition subsidies may offer interested studentsthe financial capability to obtain either an advanced degree or advanced training to gain the skills required for scientific careers involving high containment.
➢ Balancing the workload is challenging in BSL-3-Ag/BSL-4 facilities leading to workforce retention issues. In addition, working on livestock animal studies within a GLP framework can stress the highly-trained workforce, unless there is a clear recognition and funding of the additional resources and specialized capabilities required to perform this type of research to a GLP-like quality standard.

The relative global scarcity and high cost of building, maintaining and operating BSL-3-Ag and BSL-4 facilities capable of supporting One Health-related large animal research programs came up as an issue throughout the workshop, but was not focused on explicitly. The fixed operating and overhead costs of the major large animal BSL-3-Ag/BSL-4 facilities are significant, and can account for upwards of 75 percent of the total facility budget. Maintaining the staffing expertise with their specialized science readiness and operational programs that deliver regulatory, biosafety, and biosecurity compliance requires long-term strategic planning and funding commitments at national and global levels. A follow-up workshop could explore various opportunities and mechanisms through which the One Health research community could fund and more effectively leverage this costly infrastructure and expertise to establish a global ‘community of practice’ capable of responding to known and emerging high-consequence pathogens.
